# Differential microRNA expression for diagnosis and prognosis of papillary thyroid cancer

**DOI:** 10.3389/fmed.2023.1139362

**Published:** 2023-04-05

**Authors:** Viviana A. Ruiz-Pozo, Santiago Cadena-Ullauri, Patricia Guevara-Ramírez, Elius Paz-Cruz, Rafael Tamayo-Trujillo, Ana Karina Zambrano

**Affiliations:** Centro de Investigación Genética y Genómica, Facultad de Ciencias de la Salud Eugenio Espejo, Universidad UTE, Quito, Ecuador

**Keywords:** papillary thyroid cancer, miRNAs, diagnosis, biomarkers, tumorigenesis

## Abstract

Papillary thyroid cancer accounts for 85% of thyroid cancer. The diagnosis is based on ultrasound methods and tumor biopsies (FNA). In recent years, research has revealed the importance of miRNAs, non-coding RNA molecules that regulate gene expression and are involved in many diseases. The present mini review describes upregulated and downregulated miRNAs expression in papillary thyroid cancer patient samples (tissue, serum, plasma) and the genes regulated by these non-coding molecules. In addition, a bibliographic search was performed to identify the expression of miRNAs that are common in tumor tissue and blood. The miRNAs miR-146b, miR-221-3p, miRNA 222, miR-21, miR-296-5p, and miR-145 are common in both tissue and bloodstream of PTC patient samples. Furthermore, these miRNAs regulate genes involved in biological processes such as cell differentiation, proliferation, migration, invasion, and apoptosis. In conclusion, miRNAs could potentially become valuable biomarkers, which could help in the early diagnosis and prognosis of papillary thyroid cancer.

## Introduction

Thyroid cancer is one of the most frequent neoplasms, the incidence of which increases yearly. It is estimated that 586,202 new cases of thyroid cancer were diagnosed until 2020 worldwide (1). Papillary thyroid cancer (PTC) is the most common type and accounts for 85% of these cases (2). Patients diagnosed with PTC have a good prognosis; however, 10% develop metastases. The number of patients diagnosed with PTC is increasing due to improvements in diagnostic methods (3).

One of the conventional diagnostic methods is ultrasound, which detects thyroid nodules in approximately 68% of PTC patients (4). Another diagnostic method is an ultrasound-guided fine needle aspiration biopsy (FNA), which has limitations and may lead to inconclusive results (5). Therefore, establishing biomarkers that could contribute to an accurate diagnosis is essential.

microRNAs (miRNAs) are small non-coding molecules of approximately 19 to 24 nucleotides related to gene regulation. Alteration of some of these miRNAs has been associated with the expression of pathological features in PTC and gene dysregulation in PTC tumor-promoting cells (4). Hence, obtaining specific miRNA expression patterns may improve the patient’s diagnosis using less invasive methods (6).

This review aims to describe the miRNAs involved in PTC from different samples, such as blood and tumor tissue, identifying common miRNAs in both samples that could be used as biomarkers.

## The role of microRNAs in the oncogenesis of PTC

The miRNAs are small molecules involved in post-transcriptional gene expression processes (7). These molecules can bind and regulate several target messenger RNAs (mRNAs); one mRNA molecule can be regulated by different miRNAs (8). miRNAs are involved in fundamental processes such as cell signaling and homeostasis; control of these processes prevents uncontrolled cell proliferation, modulates cell differentiation, and regulates mRNAs in response to physiological processes (9).

*In vivo* and *in vitro* analyses have revealed the role of miRNAs in cancer. The miRNAs can be described as oncomiRNAs, decreasing the expression of tumor suppressor genes (10), or as suppressor miRNAs, reducing the expression of oncogenes or inducing apoptosis (8).

In papillary thyroid cancer, oncogenic and suppressor miRNAs have been described as being upregulated and downregulated. Moreover, different miRNAs may regulate the same genes related to biological processes such as angiogenesis, proliferation, and apoptosis in PTC (Table 1).

**Table 1:** Differentially expressed miRNAs and genes related to PTC.

miRNAs	Target/regulators	Number of clinical samples	Upregulated/downregulated	Prognosis	References
miR-7-5p	*RAF1,IRS1, EGFR, PIK3CB*	41 PTC tissues and 39 tissues of HC	Down	PTC proliferation and invasiveness	(4)
miR-9-5p	*BRAF*	19 RC-PTC tissues, 47 NR-PTC tissues	Down	PTC recurrence	(11, 12)
miR-29b-3p	*COL1A1, COL5A1*	48 PTC tissue and cell lines	Down	EMT, invasion, migration, and proliferation of PTC cells	(13)
miR-30b-5p	*GALNT7*	60 PTC Tissue	Down	Proliferation, invasion, and migration of PTC cells	(14)
miR-101	*ITGA3*	126 PTC tissues	Down	PTC progression	(15, 16)
miR-103	*ITGA2*	126 PTC tissues	Down	PTC progression	(15)
miR-152-3p	*MAP2K1, EGFR, KIT, ERBB3*	41 PTC tissues and 39 tissues of HC	Down	Promotes PTC development	(4)
miR-203	*AKT3*	65 PTC tissue, 40 BTN	Down	Epithelial-mesenchymal transition (EMT), migration, proliferation, invasion, and apoptosis of PTC cells	(17)
miR-299-3p	*ITGAV*	126 PTC tissues	Down	Cell progression and apoptosis	(15, 18)
miR-363-3p	*NOB1*	PTC cell line	Down	PTC cell proliferation, migration, invasion, and tumor growth	(19)
miR-369-3p	*TSPAN13*	Cell lines	Down	Proliferation, and apoptosis of PTC cells	(20)
miR-375	*ERBB2*	60 pairs of PTC and HC. Cell lines	Down	PTC cell proliferation, invasion, migration, and apoptosis	(21)
miR-532-5p	*KRAS, MKL2*	60 tissues, 70 benign nodules	Down	Tumor progression	(22, 23)
miR-1301-3p	*HMGB1, STAT3, PCNA*	30 PTC tissues	Down	Promotes cancer proliferation	(24–26)
		82 PTC tissues, 82 benign tissues			
		35 PTC tissues, 35 benign tissues			
miR-15a	*AXIN2, FOXO1*	126 PTC tissues	Up	Cell proliferation and invasion *via* promoting apoptosis	(15, 27)
miR-92a-3p	*VHL*	100 serum patients with and 96 HC	Up	Tumor progression and vascular invasion	(28, 29)
		Serum			
miR-155	*APC*	20 PTC tissues, 20 HC and cell lines	Up	PTC cell growth, migration, invasion, and apoptosis	(30, 31)
miR-181b	*CYLD*	49 PTC tissue, 23 with NG and 57 HC	Up	Cell proliferation, and apoptosis	(32)
		7 plasma samples and 7 HC (30)			
miR-206	*MET*	126 PTC tissues	Up	PTC progression	(15, 33)
miR-221-3p	*EGFR, KIT, KDR, ERBB3, PIK3R1*	41 PTC tissues and 39 tissues of HC	Up	Promotes invasiveness and migration of PTC cells	(4, 34, 35)
		174 FNAC			
		9 Rc-PTC tissues, 7 NR-PTC tissues. 78 plasma samples			
miRNA 222	*AXIN2, KIT*	9 Rc-PTC tissues, 7 NR-PTC tissues.	Up	Associated with PTC multifocality	(32, 34, 35)
		78 plasma samples			
		174 FNAC			
		49 PTC tissue, 23 with NG and 57 HC			
		7 plasma samples and 7 HC			
miR-455-3p	*CXCL12*	60 tissues, 70 BTN	Up	Metastasis of thyroid cancer cells	(22)
miR-551b-3p	*ERBB4*	60 tissues, 70 benign nodules	Up	Malignancy and progression of PTC	(4, 22, 36)
		Tissue			
miR-21	*PDCD4, THRB, VHL*	19 RC-PTC tissues, 47 NR-PTC tissues.	Down	PTC recurrence	(11, 37, 38) (39, 40)
		321 PTC tissues	Down		
		PTC cell line	Up		
				Lymph node metastasis, cell proliferation	
		58 serum patients with PTMC, 47 with PTC, 35 patients with BTN and 40 HC.	Up		
miR-25-3p	*AKT1*	56 PTC tissue, 95 BTN, 10 HC, 7 plasma samples serum	Up (before thyroidectomy)	PTC malignancy	(41, 42)
			Down (after surgery)		
miR-145	*AKT3*	PTC Tissue and Plasma samples	Down	Growth and metastasis of thyroid cancer cells	(43)
			Up		
miRNA 146b	*PIK3CB, NRAS, BRAF*	41 PTC tissues and 39 tissues of HC (4)	Up	Risk of vascular invasion and metastasis to lymph nodes and distant organs	(4, 34, 35, 44, 45)
		9 RC-PTC tissues, 7 NR-PTC tissues. 78 plasma samples (14)	Up (before thyroidectomy)		
		-	Down (after surgery)		
		174 FNAC	Up		
		19 plasma samples with benign lesions 70 plasma samples with PTC	Up		
miR-296-5p	*PLK1*	100 serum patients with and 96 HC	Up	Cell proliferation and apoptosis	(28, 46)
		28 PTC tissue and HC	Down		

PTC, Papillary Thyroid Cancer; HC, Healthy control; FNAC, fine needle aspiration cytology; RC, Recurrent; NR, Non recurrent; NG, nodular goiter; BTN, benign thyroid nodules.

## Analysis of microRNAs in tumoral tissue of PTC

Papillary thyroid cancer can be diagnosed with an ultrasound, FNA, and cytology testing. However, it is sometimes impossible to differentiate between malignant and benign tissue at a preoperative stage (4). Some investigations have revealed that the expression of specific miRNAs can be associated with characteristics such as tumor size, capsular and vascular invasion, and tumor aggressiveness (47). Rogucki and colleagues have shown that a panel of four miRNAs (miR-152-3p, miR-221-3p, miR-551b-3p, and miR-7-5p) could be used for the diagnosis of PTC. Furthermore, miR-152-3p, miR-221-3p, and miR-7-5p target *EGFR* gene regulation (4). The *EGFR* gene is a tyrosine kinase involved in cell proliferation, and mutations in this gene are associated with PTC tumor cell development (48). Qiao et al. revealed that miR-1301-3p and miR-532-5p are downregulated, whereas miR-551b-3p and miR-455-3p are upregulated in PTC patients compared with benign nodules and suggested that the four miRNAs have diagnostic value (22). The decrease in the expression of miR-1301-3p has been associated with the overexpression of the *PCNA* gene. The *PCNA* gene regulates the cell proliferation cycle (DNA transcription, synthesis, and repair), and studies reveal that overexpression of this gene promotes the differentiation and progression of various types of tumors (24). In another study, Wang et al. showed that overexpression of miR-551b, a regulator of the gene *ERBB4*, may predict poor prognosis in patients with PTC (36). For instance, *ERBB4* could be downregulated in thyroid tumors; hence, this miRNA could be considered a novel biomarker (49).

In a group of 126 tissue samples from patients with PTC, it was observed that miR-15a was overexpressed, and its target gene *FOX01* decreased in expression (15). FOXO1 is a transcription factor that regulates proliferation, differentiation, and response to cellular oxidative stress (50). The difference in its expression could trigger the development of tumor cells.

Integrin proteins are molecules involved in cell–cell and cell-extracellular matrix binding. Some integrin genes *ITGAV, ITGA3,* and *ITGA2* are regulated by miR-299-3p, miR-101, and miR-103, respectively (Table 1). Studies mention that integrins play an important role in tumor progression and metastasis (15). Mautone et al. mentioned that higher *ITGA3* expression is associated with a higher risk of tumor recurrence, advanced stage of disease, and worse prognosis. In contrast, *ITGA2* and *ITGAV* expression was associated with an intermediate risk of recurrence (51).

Moreover, in an analysis of PTC tumor samples from recurrent and non-recurrent patients, miR-9-5p was downregulated, and one of the target genes of miR-9-5p is the *BRAF* gene. The *BRAF* gene triggers processes such as extrathyroidal invasion, metastasis, and recurrent disease (52). Similarly, in another study, the authors mentioned that low expression of miR-30b-5p is associated with worse prognostic features of PTC. Furthermore, miR-30b-5p is associated with *GALNT7* gene regulation, and the microRNA may trigger cell proliferation and invasion *via* EGFR/PI3K/AKT pathway (14).

Likewise, miR-363-3 p has been associated with tumor suppression in several types of cancer (53). *In vivo* and *in vitro* investigations have associated the low expression of miR-363-3p with the overexpression levels of NOB1 (Nin-one binding protein). NOB1 silencing modifies the cell cycle, increasing the G0/G1 phase and decreasing the S phase, inhibiting PTC growth, proliferation, and invasion (19).

miRNAs like miR-375, miR-203, miR-29b-3p, and miR-369-3p were downregulated, whereas their target genes *ERBB2, AKT3, COL1A1, and TSPAN13* were overexpressed. Tumor development, metastasis, apoptosis, and cell invasion are some of the processes in which these genes are involved (13, 17, 20, 21).

## Analysis of microRNAs in blood (serum/plasma) of PTC

The expression of circulating miRNAs in some types of cancer has contributed to the development of noninvasive diagnostic tests (54). Furthermore, research reveals that different circulating miRNAs expression are released by cancer cells, accumulate in the bloodstream, could transmit cell-to-cell signals, and regulate target genes (55).

Studies establish differences between the expression patterns of microRNAs in plasma and serum. Some miRNAs are more frequently observed in serum, whereas others are more recurrent in plasma (28, 56, 57). For instance, He et al. established that miR-92a-3p, miR-145-5p, and miR-155-5p are more observed deregulated in plasma samples, whereas miR-222-3p have been more frequently observed deregulated in serum samples, in 5 types of cancer (58). The differences are probably due to the intercellular trafficking of miRNAs during the coagulation process. Thus, a more detailed understanding of the coagulation process is essential to determine its impact on the spectrum of miRNAs in serum and plasma (59).

Zou et al. screened for miRNAs in the serum of 100 patients with PTC and 96 healthy controls. The investigation revealed that a group of miR-25-3p, miR-296-5p, and miR-92a-3p were overexpressed in PTC patient samples compared to controls. Moreover, miR-25-3p, miR-296-5p, and miR-92a-3p target the genes *AKT1,* *PLK1,* and *VHL1,* respectively (Table 1) (29, 41, 46). Furthermore, the diagnostic value was assessed by the areas under the curves (AUC), and the values were statistically significant, with values of 0.727, 0.771, and 0.862, respectively. Thus, these miRNAs may be used as a noninvasive diagnostic test (28).

Research suggests that circulating levels of tumor-suppressing miRNAs are downregulated, whereas those that promote oncogenic activation are upregulated in PTC patients (55). For instance, Lee et al. found that miR-155 was upregulated in the plasma of patients with PTC with an AUC of 0.695 (44). miRNA-155 acts as an oncogenic miRNA and targets the APC gene. The dysregulation of miR-155 causes low APC expression, activating the WNT/βcatenin signaling pathway and triggering increased tumor cell viability and growth (30).

Kondrotienė et al. compared plasma samples from patients before and after thyroidectomy. The authors found that some miRNAs like miR-21 and miR-181b were overexpressed in plasma samples before surgery, whereas, after surgery, the expression of these miRNAs decreased (32). Therefore, circulating levels could be useful to differentiate the stages of the disease. In addition, miR-181b directs the *CYLD* gene, which regulates processes of the NF-κB pathway, involved in inflammatory responses in tumorigenesis (60). Similarly, the *VHL* gene is regulated by miR-21 and miR-92a-3p. The *VHL* is a tumor suppressor gene that is downregulated in some types of cancer. This gene dysregulation is involved in the development of carcinomas and clinically manifests aggressive behavior (29).

## Common miRNAs detected in tissue and blood on PTC

The expression of some tumor-deregulated miRNAs has also been found up or down regulated in blood, making them potential noninvasive markers for PTC patients (61).

Studies have described alterations in miRNA expression between tissue and blood samples (62). Zou et al. stated that the phenomenon might be partly due to the communication of miRNAs between the tumor, tumor microenvironment, and peripheral blood circulation. Additionally, different biological states of tumors, the influence of other non-tumor cells, or changing immune states could also alter the expression of miRNAs (28).

Lee et al. analyzed tissue and plasma samples from patients with PTC. The authors identified that in tissue samples, the expression of miR-222 was 10.8 times higher, and miR-146b was 8.9 times higher in tumor samples with recurrence (34). Furthermore, in the same study, the expression of miRNAs was analyzed in plasma samples from 42 PTC patients before and after surgery. It was found that miR-146b, miR-221, and miR-222 were significantly upregulated before and downregulated after surgery (34). Similarly, in a study of 100 PTC samples, it was shown that in tumors where the *BRAF* ^V600E^ mutation was detected, miR-146b was overexpressed, and it was determined in a subsequent study that this association could triggered shorter survival (63). Likewise, another study concluded that miR-146b, miR-221, and miR-222 are related to the regulation of the *KIT* gene. This gene is a receptor tyrosine kinase, which is involved in cell differentiation and growth. *KIT* plays the role of an oncogene in some types of cancer, and in PTC, its expression is lower than in normal tissues (64).

Furthermore, Boufraqech et al. analyzed the expression of miR-145 and revealed that it is downregulated in PTC tissues, whereas, in the plasma of PTC patients, it is upregulated. Furthermore, the authors demonstrated that miR-145 has tissue specific tumor suppressor functions by interacting with *AKT3*, which regulates the PI3K/Akt pathway. *In vivo* and *in vitro* studies show that the upregulation of miR-145 decreases the growth and metastasis of thyroid cancer cells (43).

**Figure 1:**
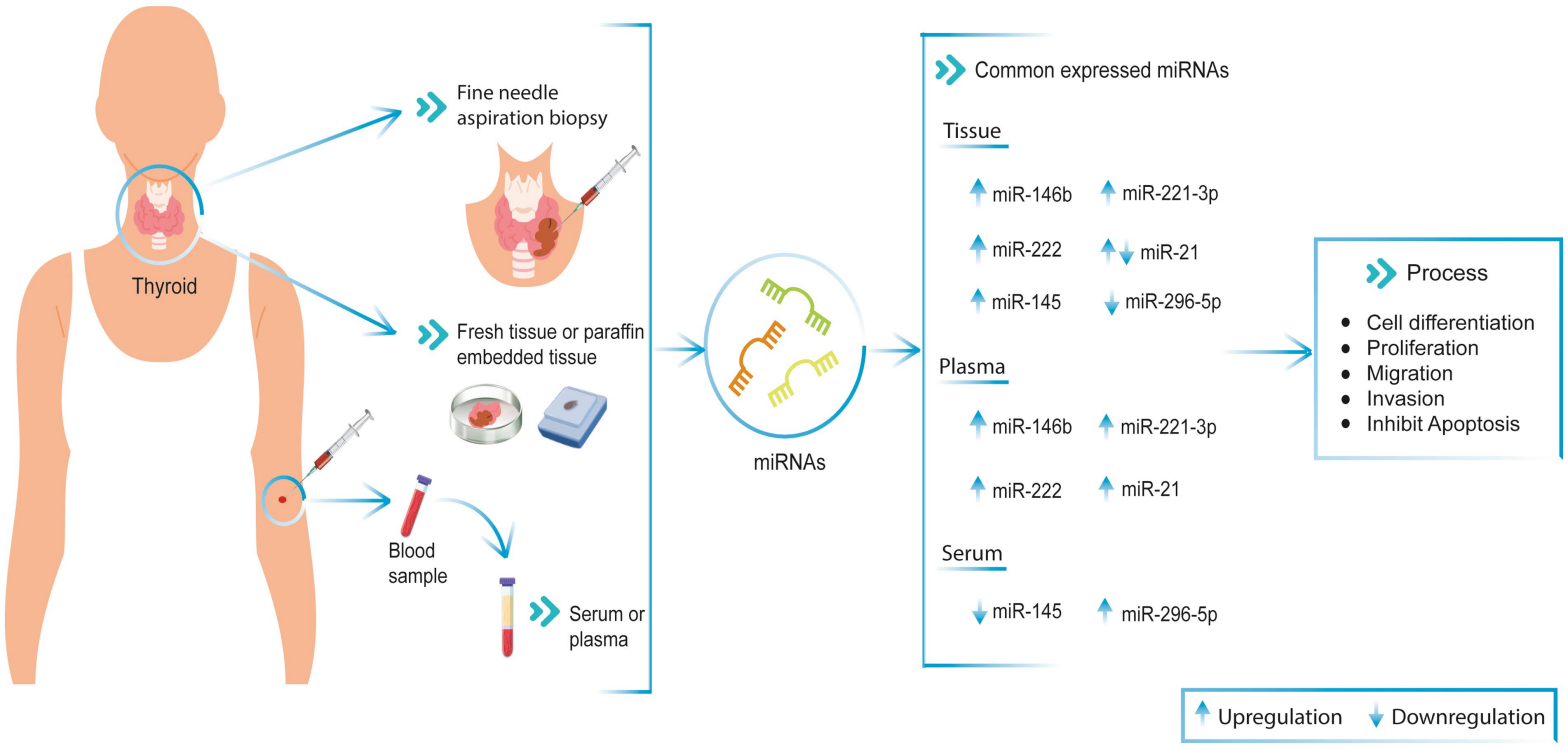
Commonly miRNAs expression in PTC patient’s tissue and blood (serum and plasma). These miRNAs are involved in cell differentiation, proliferation, migration, invasion, and apoptosis processes in PTC patient samples. **↑** Upregulated, **↓** Downregulated.

Likewise, another study with tissue samples from 28 patients with PTC and adjacent non-tumor tissue from the same patients revealed that miR-296-5p expression in tissue was downregulated (46). In contrast, Zou et al. described that plasma miR-296-5p was overexpressed in PTC patients. Therefore, based on the different levels of miR-296-5p expression between plasma and tissue, miR-296-5p may be a biomarker for the diagnosis of thyroid cancer (Figure 1) (28).

## Diagnostic utility of microRNA in PTC

Thyroid cancer presents several tumor types with different levels of differentiation and cellular origin, such as PTC (65). Research has determined that the expression levels of miRNAs are different in normal tissues compared to tumor tissues, as the role played by miRNAs is the regulation of signaling pathways. These miRNAs have been related to cell proliferation, survival, invasion, and migration processes. In other words, miRNAs could be valuable tools for determining the cellular characteristics of tumors (66, 67).

Additionally, the expression of miRNAs can give information about the early stages of the disease by analyzing circulating miRNAs in blood (68).

The technologies used to determine the expression of miRNAs have been diverse. Early methods were based on hybridizing single or multiple miRNAs and panel development using microarray platforms. Nowadays, Next Generation Sequencing (NGS) is being applied to obtain gene expression profiles. NGS makes it possible to get information on coding and non-coding RNAs to analyze the expression of specific miRNAs (67). Despite the more accurate technologies currently available, it is still necessary to work on variables such as sample size, sample type, and sample collection conditions to obtain miRNAs. In addition, it is important to evaluate the expression levels of miRNAs in both blood and tissue that may be used as biomarkers in PTC diagnosis and prognosis.

## Conclusion

The incidence of thyroid cancer has increased worldwide (69). Fine needle aspiration (FNA) biopsy is one of the conventional diagnostic methods; however, it is an invasive test, and its results could be indeterminate (70).

The use of miRNA expression has been investigated as an alternative non-invasive diagnostic method in different types of cancer. miRNA molecules are released from the tissue into the bloodstream at separate stages of the disease (10).

In this review, we collected miRNA expression data in fresh tissue, FNA samples, and blood (serum/plasma) from patients with PTC (Table 1). In addition, common miRNAs in both tissue and blood, and their relationship with genes associated with physiological processes, were described. The common miRNAs identified in this work are miR-146b, miR-221, miR-222, miR-21, miR-145, and miR-296-5p in patients with PTC. These miRNAs are involved in cell differentiation, proliferation, migration, invasion, and apoptosis processes (45, 71). Moreover, it is important to mention that some studies use miRNAs panels to differentiate between malignant and benign tissues (4, 22).

In conclusion, this literature research provided data on miRNA expression in tissue and blood (serum/plasma) that could be used as biomarkers for diagnosis, and prognosis in patients with PTC.

## Author contributions

VR-P, SC-U, PG-R, and AZ: conceptualization and writing—review and editing. EP-C and RT-T: research. AZ: supervision and conceptualization. All authors contributed to the article and approved the submitted version.

## Funding

The publication fee will be funded by Universidad UTE.

## Conflict of interest

The authors declare that the research was conducted in the absence of any commercial or financial relationships that could be construed as a potential conflict of interest.

## Publisher’s note

All claims expressed in this article are solely those of the authors and do not necessarily represent those of their affiliated organizations, or those of the publisher, the editors and the reviewers. Any product that may be evaluated in this article, or claim that may be made by its manufacturer, is not guaranteed or endorsed by the publisher.
